# “It makes me feel not so alone”: features of the Choose to Move physical activity intervention that reduce loneliness in older adults

**DOI:** 10.1186/s12889-021-10363-1

**Published:** 2021-02-06

**Authors:** Thea Franke, Joanie Sims-Gould, Lindsay Nettlefold, Callista Ottoni, Heather A. McKay

**Affiliations:** 1 Active Aging Research Team,, The University of British Columbia, Vancouver, BC Canada; 2 Department of Family Practice, Faculty of Medicine, The University of British Columbia,, Vancouver, BC Canada

**Keywords:** Isolation, Loneliness, Older adults

## Abstract

**Background:**

Despite the well-known health benefits of physical activity (PA), older adults are the least active citizens. Older adults are also at risk for loneliness. Given that lonely individuals are at risk for accelerated loss of physical functioning and health with age, PA interventions that aim to enhance social connectedness may decrease loneliness and increase long-term PA participation. The objectives of this mixed-method study are to: (1) evaluate whether an evidence-based PA intervention (Choose to Move; CTM) influenced PA and loneliness differently among self-identified ‘lonely’ versus ‘not lonely’ older adults and (2) to describe factors within CTM components most likely to promote social connectedness/reduce loneliness.

**Methods:**

CTM is a flexible, scalable, community-based health promoting physical activity intervention for older adults. Two community delivery partner organizations delivered 56 CTM programs in 26 urban locations across British Columbia. We collected survey data from participants (*n* = 458 at baseline) at 0 (baseline), 3 (mid-intervention) and 6 (post-intervention) months. We conducted in depth interviews with a subset of older adults to understand how CTM facilitated or impeded their PA and social connectedness.

**Results:**

PA increased significantly from baseline to 3 months in lonely and not lonely participants. PA decreased significantly from 3 to 6 months in lonely participants; however, PA at 6 months remained significantly above baseline levels in both groups. Loneliness decreased significantly from baseline to 3 and 6 months in participants identifying as lonely at baseline. Factors within CTM components that promote social connectedness/reduce loneliness include: *Activity coach characteristics/personality traits and approaches; opportunity to share information and experiences and learn from others; engagement with others who share similar/familiar experiences; increased opportunity for meaningful interaction;* and *accountability.*

**Conclusion:**

Health promoting interventions that focus on PA and social connectedness through group-based activities can effectively reduce social isolation and loneliness of older adults. Given the ‘epidemic of loneliness’ that plagues many countries currently, these kinds of interventions are timely and important. Research that further delineates mechanisms (e.g., sharing experiences vs. lectures), that modify the effect of an intervention on social connectedness outcomes for older adults engaged in community-based PA programs would be a welcome addition to the literature.

## Background

In the next decade, we will experience an unprecedented escalation in the number of older adults in most developed countries worldwide--with an anticipated rise in mobility loss [1], physical inactivity [2] and loneliness [3]. In Canada, at least four of five older adults do not meet Canadian Physical Activity Guidelines of 150 min of moderate-to-vigorous physical activity (PA) per week [4]. Developed countries have described an ‘epidemic of loneliness’ sweeping major cities [5]. In the United Kingdom loneliness was identified as a key public health priority by appointing a ‘Loneliness Minister’, implementing a loneliness strategy for Scotland [6] and launching its ‘Campaign to End Loneliness’ (www.campaigntoendloneliness.org) which targets older adults. Loneliness is closely linked with accelerated loss of physical functioning and health and age [7, 8], thus, taking action to counter loneliness is timely and important. Interventions designed to increase long-term PA participation and promote social connectedness [9] may effectively stop or slow mobility loss [10] and diminish loneliness [11–13]. However, interventions that aim to positively promote social connectedness and reduce feelings of loneliness among older adults are often grouped with interventions that promote social contact/ reduce isolation (related but distinct concepts) [14–17]. Differentiating the distinct effect of an intervention, and in particular a PA intervention, on social connectedness/loneliness versus social contact/isolation has for the most part been overlooked.

Loneliness and social connectedness are positioned at opposite ends of a continuum. If an individual is lonely, then they are not socially connected. If they are socially connected, then they are not lonely. *Lonelines*s is a perceived lack in quality or quantity of one’s relationships [18] and predicts various health outcomes, including: systemic inflammation, increased blood pressure, depression, weight gain, smoking alcohol/drug use, physical inactivity, and alone time [19–24]. *Social connectedness* is defined as feelings of interpersonal connection and meaningful, close, and constructive relationships with others (i.e., individuals, groups, and society). A socially connected person feels that they: (i) care about others and are cared about by others, and (ii) belong to a group or community [12]. Caring and respect in social relationships prompts a sense of well-being—together they act as a buffer against the various health outcomes [e.g., high blood pressure, heart disease, a weakened immune system and cognitive decline] associated with loneliness [19, 20]. Social connectedness (e.g., social bonding) contributes to older adults’ engagement and acceptability of PA interventions [25]. Yet most physical activity interventions, for community dwelling older adults, fail to create and sustain social connectedness among participants [26].

Loneliness must be distinctly defined from *social isolation* which is a reduced social network [27, 28]. It is a quantifiable measure of the number and structure of one’s relationships (i.e., social, family, and friend contacts) or frequency of interaction with others (i.e., social contact). *Social contact* is described as physical closeness, interaction (face-to-face/ in-person, internet-based, and/or telephone) or touch encounters [29] with others [30, 31]. Social isolation/social contact are objective constructs whereas loneliness/social connectedness are subjective. To illustrate, an older person may be alone (i.e., isolated) but still feel a sense of social connectedness. Conversely, they may be surrounded by people (i.e., have social contact) but still feel lonely [32, 33]. Although isolation/social contact may influence social connectedness/loneliness [34], they are not necessary mediators [35].

The distinction is important when designing PA interventions that promote social connectedness/ reduce loneliness. Specific mechanisms of an intervention (e.g., goals, components, activities, mode and dose of delivery) will vary if the specific goal is to positively affect loneliness/social connectedness outcomes versus isolation/social contact outcomes [34]. For example, rather than bringing older adults together for informative lectures (isolation/social contact), interventions may offer activities that create social bonding such as storytelling/sharing (loneliness/social connectedness). However, few studies (i) clearly describe the mechanisms of the intervention, and (ii) assess the extent to which the hypothesized mechanisms map on to factors within those mechanisms that may promote social connectedness/reduce loneliness [12].

### Context

**Fig. 1:**
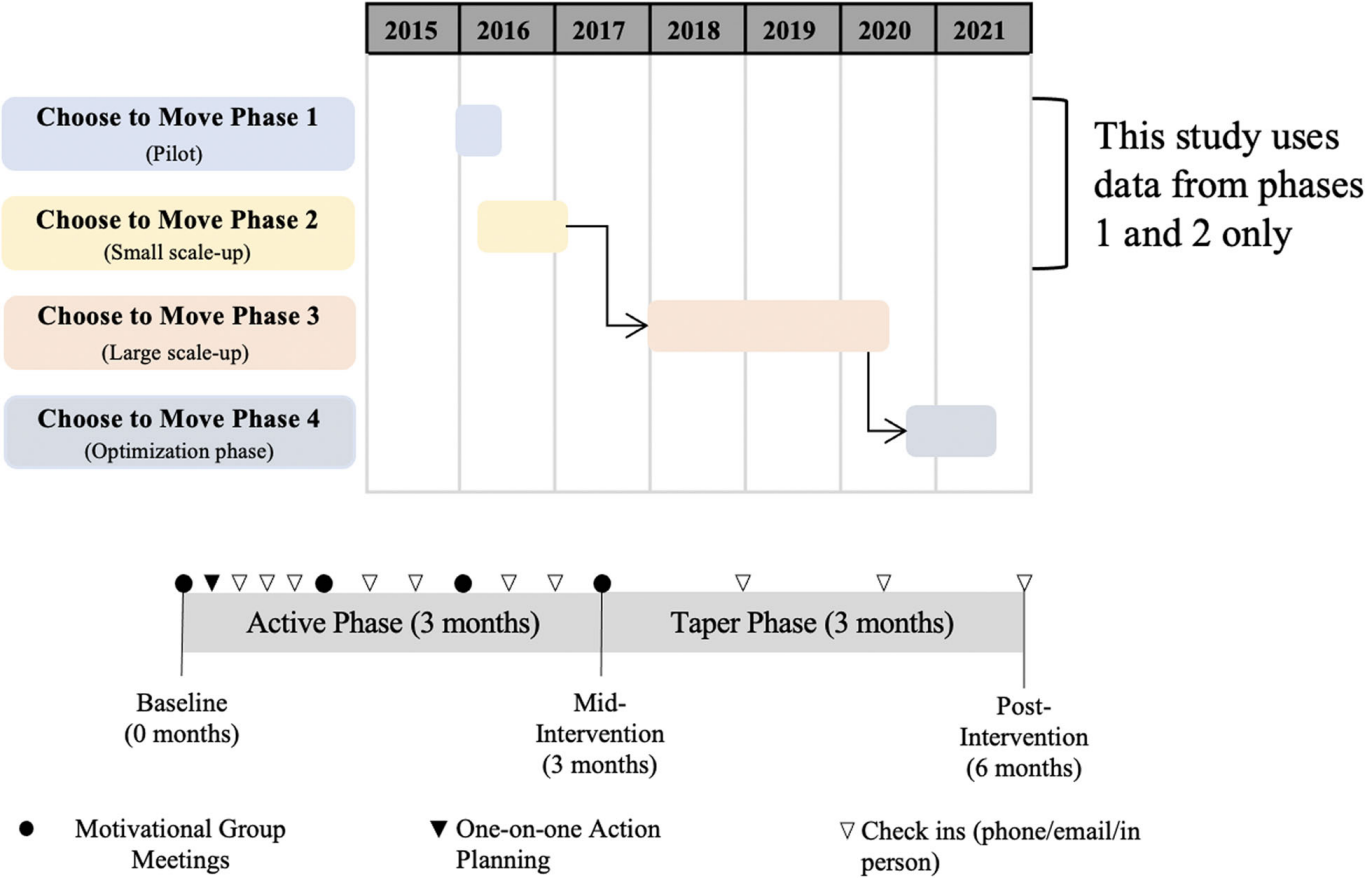
The upper portion illustrates the phased scale-up of Choose to Move. Black arrows between phase 2 and 3, and between phase 3 and 4 indicate the formal and systematic adaptation of the model to enhance fit and optimize the model. We use data from phases 1 and 2 in this manuscript. The lower panel illustrates the time points for one Choose to Move program. Data collection (surveys - all participants; interviews - subset) occurred at 0, 3 and 6 months. Lower panel is adapted with permission from “Implementation of a co-designed physical activity program for older adults: positive impact when delivered at scale,” by McKay H, Nettlefold L, Bauman A, Hoy C, Gray SM, Lau E, and Sims-Gould J, 2018, BMC public health, 18 [1]:1289. CC BY 4.0

In partnership with British Columbia (BC) Ministry of Health, we co-created a community-based, flexible, scalable health promoting PA and social connectedness intervention called Choose to Move [36]. In collaboration with key community stakeholders, CTM is being scaled up in a phased manner across the province of BC, Canada [2016–2021; Fig. 1]. We used phase 1 and phase 2 (small scale up) data for the current study (Jan 2016- May 2017). In phases 1 and 2 (Jan 2016-May 2017) CTM effectively enhanced PA, mobility and social connectedness, and reduced social isolation in older adults [37]. Our implementation evaluation demonstrated that CTM could be effectively adapted to context [38] and implemented at scale by trained activity coaches in collaboration with key community recreations organizations with established reach to older adults [37, 38].

## Aims and objectives

The aim of our mixed-method study is to evaluate whether older adults’ perceptions of loneliness modify the effect of CTM on PA and social connectedness outcomes.

Our specific objectives are twofold: (i) to evaluate whether CTM influenced PA and loneliness differently among older adults who identified as lonely versus older adults who identified as not lonely at baseline; (ii) to describe factors within CTM components most likely to promote social connectedness/reduce loneliness.

## Methods

### Study design and setting

#### Choose to move

Elsewhere we described the CTM intervention, implementation and evaluation frameworks that guide our work [36, 37, 39], the benefits of CTM on PA and social connectedness [37] and factors that influenced implementation [38]. Briefly, CTM is a 6-month, choice-based health promotion program that supports older adults with low levels of PA to become more physically active and socially connected. Development of CTM was informed by the CHAMPS intervention [40] based on its implementation and successful dissemination at an organizational level [36]. CHAMPS was based on principles derived from social cognitive theory; CTM is guided by many of these same principles. In Phases 1 and 2 (Jan 2016-May 2017; the focus of this manuscript), CTM consisted of: (i) a one-on-one consultation with an activity coach, plus (ii) regular phone call ‘check-ins’ with an activity coach, and (iii) regular motivational group meetings with other CTM participants (up to 12 participants/group), led by an activity coach. Group meetings included opportunities to share experiences, educational content on a specific topic and sharing of resources. Activity coaches received [in-person] standardized CTM training. Delivery ‘dose’ of program components was greater in the first 3 m (*active phase:* one-on-one, seven phone calls, four group meetings) and tapered off in the last 3 m (*taper phase:* three phone calls; Fig. 1).

During the one-on-one consultation personal PA goals were set, action plans were created, barriers to participation were problem solved, social support was received, and PA and health-related information was provided. The personalized action plan aligned with each participants’ available resources, interests, abilities, and income. Activity coaches facilitated groups on a monthly basis (4 × 1 h each). During the group meetings, information designed to promote PA and support the development of relationships (e.g., support, companionship) among group members was presented [37]. Participants were given the opportunity to participate in group or paired discussions, shared their experiences and connected with others. Individual phone check-ins provided opportunities for activity coaches to discuss progress and setbacks and to adjust their action plans accordingly.

We describe our study design and implementation approach in detail elsewhere [36–39]. Briefly, we conducted a type 2 hybrid effectiveness-implementation study, using both quantitative and qualitative methods [41]. We measured participants at 0 (baseline), 3 (mid-intervention) and 6 (immediately post-intervention) months (Fig. 1). In phases 1 and 2 (Jan 2016-May 2017), two partner organizations delivered 56 CTM programs in 26 small (population 1000–29,999; *n* = 8 community sites), medium (population 30,000-99,999; *n* = 7 community sites) and large (population 100,000+; *n* = 11 community sites) urban communities (Statistics Canada, 2017).

#### Participants

We received consent from 458 of 534 (86%) CTM participants to participate in the evaluation. To register for CTM, participants had to be over the age of 60, speak English and engage in < 150 min/week of PA [2]. Participants had no contraindications to participate in PA based on the Physical Activity Readiness-Questionnaire+ [42], or physician clearance. CTM recruitment strategies included printed materials (e.g., community centre program guides, posters, newspaper advertisements), information sessions, radio and social media advertisements, and word of mouth [37].

### Measurements

#### Quantitative

Participants enrolled in the evaluation provided survey data at 0, 3 and 6 months. At 0 and 3 months we collected participant data at Motivational Group Meetings (or by mail if they missed a group meeting). At 6 months, participants received and returned surveys via mail [37].

##### Demographic characteristics

At baseline participants provided the following demographic data: age (as age category; 60–74, ≥75 years), sex (male, female), height and weight (used to calculate body mass index (kg/m^2^); categorized as (< 30, ≥30 kg/m^2^, level of education (secondary school or less, at least some trade/technical school or college, at least some university), ethnicity (Asian, white, other), number of chronic diseases (0, 1, ≥2), self-rated health (very poor, poor, fair, good, excellent), self-efficacy for increasing PA and accessing recreation centre services (1 item each; not at all, slightly, moderately, quite or very confident), social support for PA received from family or friends (1 item each; yes, no, not sure) and capacity for mobility (no/any difficulty walking 400 m and/or climbing one flight of stairs [43].

##### Physical activity

We assess PA using a single item questionnaire: “In the past week, on how many days have you done a total of 30 minutes or more of physical activity, which was enough to raise your breathing rate? This may include sport, exercise, and brisk walking or cycling for recreation or to get to and from places, but should not include housework or physical activity that may be part of your job” [44]. This questionnaire is reproducible [44] and shows acceptable concurrent and criterion validity [44, 45].

##### Loneliness

We used a three item questionnaire (LQ-3) with a 3 point scale to assess loneliness [46]. Questions asked were, “how much of the time do you feel (i) you lack companionship; (ii) left out; (iii) isolated from others” (3 = often, 2 = some of the time, 1 = hardly ever). The overall score reflects the sum of the three items (range 3–9) with lower scores indicating lower levels of loneliness. We classified participants as “lonely” if they responded “some of the time” or “often” to any of the 3 components on the questionnaire and as “not lonely” if they responded “hardly ever (or never)” to all 3 components [46]. This short questionnaire shows good internal consistency, discriminant validity and convergent validity [46].

##### Social isolation

We assessed isolation using three items adapted from two questions focused on frequency of contact [47]. Questions asked were “How often do you (i) get together with friends, neighbours or relatives, and do things like go out together or visit in each other’s homes?; (ii) talk on the telephone or exchange emails with friends, neighbours or relatives? and; (iii) attend meetings or programs of groups, clubs or organizations that you belong to?”. Response options are: never; less than once a month; about once a month; 2 or 3 times a month; once a week and; more than once a week (scored on a 6-point scale from 0 to 5, respectively). The 3 items are summed to create an overall social isolation score (range 0–15); lower scores indicate greater levels of social isolation. We classified participants as “socially isolated” if they reported low levels of interpersonal interaction (once/month or less) [48]. We defined that operationally as those who answered once/month or less (i.e., a score of 0, 1 or 2) for all 3 questions. As an adapted measure, psychometric properties for this in-house measurement scale are not available.

### Analysis

#### Quantitative

We used Stata v13.1 for all quantitative analysis. We first assessed differences in socio-demographic characteristics between participants who identified as lonely versus not lonely at baseline using Chi-squared or Fisher’s exact test (categorical variables: sex, age category, ethnicity, education, chronic conditions, mobility limitations) and unpaired t-tests (continuous variables: BMI).

To assess whether PA and loneliness differed by participants’ loneliness status at baseline, we used general linear mixed effects models with time (0, 3, 6 months) as a categorical predictor [37]. In model 1 we included sex and baseline loneliness (dichotomous measure; lonely vs. not lonely) as fixed effects. In model 2 we included additional covariates (age category, delivery partner, social isolation category, baseline mobility limitation, number of chronic conditions, level of education and BMI category) sequentially, testing for interactions with time after each addition. We retained the interaction of baseline loneliness*time in the model regardless of significance; other interactions were only retained if they significantly improved model fit (likelihood ratio test [49] of *p* < 0.05). We used residual plots to assess model fit and calculated adjusted values at each time point within Stata (margins command with Bonferroni adjustment to account for multiple comparisons between and within loneliness groups) [37]. We did not use any imputation techniques to address missing data.

### Data collection

#### Qualitative

We conducted in depth semi-structured audio-recorded interviews by phone with a subset of older adults at baseline (*n* = 43), 3 months (*n* = 38) and 6 months (*n* = 19). The same participants were interviewed at each time point. Participants were randomly selected from those who consented to the interview component. Interviews took approximately 15–30 min. If interview participants dropped out, the Research Coordinator asked why they withdrew and about barriers to participation. Questions included in the interview guide were developed by our research team. Interview topics included feedback on the three CTM components (one-on-one consultation, motivational group meetings, check-ins); we identified factors within these components that facilitated/impeded their influence on PA and social connectedness (e.g., meeting content, number of meetings and check-ins, perceptions of activity coach), as well as facilitators and barriers to committing and adhering to their Action Plan; continuing PA after CTM (Table 1). We fully transcribed each interview verbatim.

**Table 1:** Sample participant interview questions

Baseline	3-month follow up	6-month follow up
Why did you decide to join this program?	How is this program working for you?	How did the last three months of Choose to Move go for you?
What (if anything) is appealing to you about this program?	What are your favourite parts of the program?	How did you find the telephone check-ins?

### Analysis

#### Qualitative

We reviewed transcripts using a deductive framework analysis; framework analysis is well suited to research that has specific questions, a pre-designed sample and theoretically deduced issues [50]. In deductive framework analysis, the categories/codes are often pre-defined (e.g., by specific areas of interest to the project). Our categories/codes were created in order to systematically capture participants descriptions of (i) CTM components, (ii) factors within these components most likely to promote social connectedness/reduce loneliness and (iii) any social connectedness indicators [12] linked to those factors. There are 7 stages to framework analysis [50–52]. We briefly discuss each stage below. First, the lead author received the transcripts (stage 1- transcription), read through the transcripts to become more familiar with the interviews (stage 2 - familiarize). Even though we had pre-defined categories/codes we still did open coding on a few transcripts to ensure any codes were not missed (stage 3 – coding). We held a series of team meetings to discuss the framework (stage 4 – developing a framework) and then the lead author coded the remainder of the transcripts using the framework and added any additional codes if missing from the framework (stage 5- applying the framework). The lead author coded full paragraphs to not lose contextual meaning. We adopted the constant comparison method [53] to look for patterns and connections within and between cases and codes and within and across groups. This revealed similarities and differences in the data. We compared responses between participants who were lonely with participants who were not lonely (stage 6- charting). We then began interpreting the data by mapping connections between codes to explore relationships and develop themes within each category.

We used a number of strategies to reinforce the rigor of our study. They were: cross-checking full transcripts against original audio files for quality and completeness; “member reflections” which involve the process of re-iterating interpretations of what was heard during the interview back to participants in order to avoid misunderstanding. We also conducted reflexive memoing throughout data generation and data analysis processes [54]. We also created an audit trail to record all key procedural and analytical decisions made throughout the study [55, 56].

## Results

### Quantitative

#### Participants

Our final sample size at baseline was 452 participants [6 participants did not complete baseline surveys]. We summarize socio-demographic characteristics for the whole sample in Table 2 and for the interview subset in Table 3. As previously reported [37], in the whole sample most participants identified as women (77%), lived in medium to large urban centres (77%), had at least some post-secondary education (75%), no mobility limitations (57%) and identified as white (86%). Specific to these analyses, 58% of participants identified as lonely at baseline (*n* = 261). Those who identified as lonely were more likely to be women and reported lower self-rated health compared to same-age peers (Table 2). Less than 1% of participants (*n* = 4) identified as socially isolated at baseline. Given the low prevalence of socially isolated participants in this group we were unable to include social isolation in the models; therefore, we focused solely on loneliness. Among participants who dropped out of CTM (*n* = 49), withdrew from the evaluation (n = 2) or missed an evaluation timepoint (*n* = 51), the proportion who identified as lonely was similar between those who withdrew from, and those who remained in the study (58%).

**Table 2:** Baseline socio-demographic characteristics in participants classified as ‘lonely’ vs. ‘not lonely’

	Not lonely	Lonely	Total
**Participants,** ***n*** **(women/men)**	191 (138/53)	261 (212/49)*	452 (350/102)
**% (men)**	28%	19%	23%
**Age category,** ***n*** **(%)**			
60–74 years	126 (66%)	193 (74%)	319 (71%)
≥ 75 years	65 (34%)	68 (26%)	133 (29%)
**Self-reported BMI, kg/m**^**2**^			
Men (*n* = 102)	28.8 (4.6)	29.1 (4.3)	28.9 (4.5)
Women (*n* = 342)	29.0 (6.5)	29.8 (7.7)	29.5 (7.2)
**Ethnicity,** ***n*** **(%)**			
White	168 (88%)	220 (84%)	388 (86%)
Asian	15 (8%)	20 (8%)	35 (8%)
Other	8 (4%)	21 (8%)	29 (6%)
**Educational attainment,** ***n*** **(%)**			
Secondary or less	58 (30%)	55 (21%)	113 (25%)
Some trade, technical school or college	63 (33%)	87 (33%)	150 (33%)
Some university	70 (37%)	119 (46%)	189 (42%)
**Chronic Conditions,** ***n*** **(%)**			
0	28 (15%)	33 (13%)	61 (14%)
1	78 (41%)	105 (40%)	183 (40%)
≥ 2	85 (45%)	123 (47%)	208 (46%)
**Mobility limitations (walk or stair),** ***n*** **(%)**			
Yes	82 (43%)	113 (43%)	195 (43%)
No	109 (57%)	148 (57%)	257 (57%)
**Self-rated health,** ***n*** **(%)** ^**a**^			
Very poor, poor or fair for age	71 (37%)	133 (51%)	201 (45%)
Good or excellent for age	120 (63%)	128 (49%)*	248 (55%)
**Self-efficacy for increasing PA,** ***n*** **(%)** ^**a**^			
Not at all, slightly or moderately confident	79 (41%)	127 (49%)	206 (46%)
Quite or very confident	112 (59%)	134 (51%)	246 (54%)
**Self-efficacy for rec centre access,** ***n*** **(%)** ^**b**^			
Not at all, slightly or moderately confident	48 (31%)	79 (36%)	127 (34%)
Quite or very confident	105 (69%)	141 (64%)	246 (66%)

Values are *n* (%) or mean (SD). Sample sizes vary between each variable due to missing data. *Difference between groups

**Table 3:** Baseline socio-demographic characteristics for participants who were interviewed, separated by ‘lonely’ vs. ‘not lonely’

	Not lonely	Lonely	Total ^**a**^
**Participants, n (women/men)**	16 (8/8)	26 (19/7)	43 (27/16)
**% (men)**	50%	27%	37%
**Age category,** ***n*** **(%)**			
60–74 years	14 (88%)	18 (69%)	33 (77%)
≥ 75 years	2 (13%)	8 (31%)	10 (23%)
**Self-reported BMI, kg/m**^**2**^			
Men	29.4 (3.8)	29.4 (2.7)	29.6 (3.2)
Women	31.1 (6.8)	28.5 (7.5) ^b^	29.3 (7.3) ^b^
**Ethnicity,** ***n*** **(%)**			
White	14 (88%)	21 (81%)	36 (84%)
Asian	1 (6%)	2 (8%)	3 (7%)
Other	1 (6%)	3 (12%)	4 (9%)
**Educational attainment,** ***n*** **(%)**			
Secondary or less	4 (25%)	4 (15%)	8 (19%)
Some trade, technical school or college	7 (44%)	8 (31%)	15 (35%)
Some university	5 (31%)	14 (54%)	20 (47%)
**Chronic Conditions,** ***n*** **(%)**			
0	1 (6%)	4 (15%)	5 (12%)
1	5 (31%)	7 (27%)	12 (28%)
≥ 2	10 (63%)	15 (58%)	26 (60%)
**Mobility limitations (walk or stair),** ***n*** **(%)**			
Yes	7 (44%)	12 (46%)	20 (47%)
No	9 (56%)	14 (54%)	23 (53%)
**Self-rated health,** ***n*** **(%)** ^**a**^			
Very poor, poor or fair for age	12 (75%)	13 (50%)	26 (60%)
Good or excellent for age	4 (25%)	13 (50%)	17 (40%)
**Self-efficacy for increasing PA,** ***n*** **(%)**			
Not at all, slightly or moderately confident	8 (50%)	14 (54%)	22 (51%)
Quite or very confident	8 (50%)	12 (46%)	21 (49%)
**Self-efficacy for rec centre access,** ***n*** **(%)** ^**c**^			
Not at all, slightly or moderately confident	4 (31%)	11 (47%)	15 (42%)
Quite or very confident	9 (69%)	12 (52%)	21 (58%)

Values are *n* (%) or mean (SD)

^a^ one participant was missing baseline data for the loneliness questionnaire and cannot be represented in the not lonely/lonely columns. They are represented in the ‘total’ column

^b^ *n* = 26 women (one woman missing self-reported BMI)

^**c**^ *n* = 36 total

#### Physical activity

Results were similar for minimally and fully adjusted models, thus we focus on the fully adjusted model here (Table 4). At baseline, PA levels were similar between participants who identified as lonely and not lonely (mean difference: − 0.2 days/week (95% CI, − 0.6, 0.3). PA increased significantly during the active intervention phase (baseline to 3 months) in both lonely and not lonely participants. PA decreased significantly from 3 to 6 months (in the taper phase) in lonely participants only. However, PA at 6 months remained significantly above baseline levels in both groups.

**Table 4:** Outcome measures by time point and baseline loneliness category

	Month (# obs)	Not Lonely	Lonely	*p*-value (not lonely) 0–3 mo. 0–6 mo.	*P* value (lonely) 0–3 mo. 0–6 mo.
Physical activity (# days/week > 30 min)	0 (*n* = 443)	2.4 (2.1, 2.7)	2.2 (2.0, 2.4)		
	3 (*n* = 369)	3.8 (3.5, 4.1)	3.7 (3.4, 3.9)	*p* < 0.001	*p* < 0.001
	6 (*n* = 361)	3.4 (3.1, 3.7)	3.3 (3.1, 3.6)*	*p* < 0.001	*p* < 0.001
Loneliness (score; range 3–9)	0 (*n* = 442)	3.0 (2.9–3.2)**	5.7 (5.5, 5.8)		
	3 (*n* = 367)	3.2 (3.0, 3.4)**	4.8 (4.6, 4.9)	*p* = 0.2	*p* < 0.001
	6 (*n* = 357)	3.3 (3.1, 3.5)**	4.9 (4.7, 5.1)	*p* = 0.006	*p* < 0.001

Values are mean (95% CI)

Statistical models include: age category, gender, delivery organization, baseline mobility, number of chronic conditions, education and BMI category. Physical activity model additionally included statistically significant interactions of age category and number of chronic conditions with time

*Significantly different from 3 months within lonely group

**Significant between-group difference

#### Loneliness

Results were similar for minimally and fully adjusted models, thus we focus on the fully adjusted model here (Table 4). By definition, loneliness scores at baseline were significantly different between participants identifying as lonely and not lonely; this significant between-group difference was maintained at 3 and 6 months. Loneliness decreased significantly from 0 to 3 months in participants who identified as lonely at baseline; lower loneliness scores were maintained at 6 months (significantly different from baseline). There was no change in loneliness from 0 to 3 months in the ‘not lonely’ group. However, loneliness increased significantly in this group at 6 months compared to baseline.

#### Qualitative

Our deductive framework analysis consisted of three CTM intervention components as our categories. Below, we present each category and describe the themes (e.g., factors), within each CTM intervention component, found to promote social connectedness/ reduce loneliness. We highlight the social connectedness indicators of each theme in Table 5. Interview participants were men (*n* = 16) and women (*n* = 27), aged 60–74 (*n* = 33) or 75+ (*n* = 10) who identified as lonely (*n* = 26), not lonely (n = 16) or did not respond (n = 2). We present participant responses by time point (baseline, 3 months and 6 months), and whether participants identified as lonely or not lonely. Compared with not lonely participants, lonely participants more often discussed social connectedness factors (e.g., social activities, chatting) within each intervention component. Not lonely participants placed more emphasis on education and goal commitment. We focused our analysis on describing factors within CTM intervention components that may promote social connectedness/ reduce loneliness. The following themes were found: *activity coach characteristics/personality traits and approaches; opportunities to share information and experiences and learn from others; engage with others who share similar/familiar experiences; increased opportunity for meaningful interaction; and accountability.*

**Table 5:** Social connectedness features and indicators of Choose to Move’s three delivery components

Delivery Component	Feature	Social Connectedness Indicators		
		**Feeling cared for**	**Meaningful relationships**	**Feelings of belonging**
**One-on-One Consultation**	Activity Coach characteristics/personality traits and approaches	X	X	
**Motivational Group Meetings**	Activity Coach characteristics/personality traits and approaches	X	X	
	Opportunities to share information and experiences and learn from others	X	X	X
	Engage with others who share similar/familiar experiences			X
	Increased opportunity for meaningful interaction		X	X
**Check-Ins**	Activity Coach characteristics/personality traits and approaches	X	X	
	Accountability		X	

#### One-on-one consultation

Participants deemed the activity coach as essential to influencing social connectedness within all three CTM intervention components. Participants described distinct activity coach characteristics/personality traits and approaches that promote social connectedness/ reduce loneliness.

*Activity coach characteristics/personality traits and approaches*: being personable (easy to talk to), positive, accommodating, accepting, observant, careful, motivating, and approachable, offering encouragement and providing accountability.

Participants enjoyed being able to connect with an activity coach during the one-on-one consultation, and work with the activity coach to design a personalized action plan. This process enacted feelings of being listened to and cared for and supported development of a meaningful relationship between participants and their activity coach. The activity coach-participant relationship spurred feelings of motivation and encouragement.

(Not lonely, baseline)

Oh, I really like it because it’s designed individually for me and [name of Activity Coach] is really easy to talk to and very personable. And so, yeah, when we had our one-hour session on Thursday where we discussed and made the plan for this coming week. And so when she said would you be interested, she was full of ideas. And she was good at taking my ideas and adjusting them. It’s personalized. So, yeah, which made it very manageable.

(Not lonely, mid-intervention)

Yeah, oh, so for the Choose to Move, yeah, having to be accountable, that’s an important thing for me, I find that once I make the commitment and I just-- really didn’t want to disappoint anyone else, as well as myself.

#### Motivational group meetings

Motivational Group Meetings were overwhelmingly considered of great value to create and sustain social connections. The following factors within the Motivational Group Meetings were found to promote social connectedness/reduce loneliness: *activity coach characteristics/personality traits and approaches; opportunities to share information and experiences and learn from others; engage with others who share similar/familiar experiences; and increased opportunity for meaningful interaction.*

*Activity coach characteristics/personality traits and approaches*: being positive, engaging, accommodating, accepting, observant, careful, motivating, offering encouragement and calls participants by name.

(Lonely, mid-intervention)

Yeah, ‘cause when I missed one of the classes everybody said, oh, good to see you back. But they didn’t say “[name of participant].” But by the end of-- when (activity coach) said [name of participant], tell us what you’ve done, by the end of the class everybody goes, see you the next month [name of participant], right.

(Lonely, post-intervention)

She [activity coach] is so positive and she’s so encouraging. And she really knows her stuff. Because she really tries to engage everybody in-- she knows everybody and she knows everybody’s progress and ability. She is encouraging that way ‘cause she knows when someone is taking it slower ‘cause-- sprained ankle or not feeling well that day. And so she does that in the CTM too where she, you know, like, caters it kind of individually and often as a group. And it’s really hard to explain. But you do feel like you’re getting individual attention even though you’re also in a group getting to know everybody else.

*Opportunities to share information and experiences and learn from others* promoted interactions between participants, encouraged the exchange of phone numbers, provided personal introductions, and engaged participants in paired and group discussions to share information on community resources.

Participants discussed how they enjoyed activities that offered them the opportunity to share information and experiences with other group members. Being able to learn from others created a sense of bonding, belonging and being cared for, and developed meaningful relationships.

(Lonely, mid-intervention)

Well, everybody got to share what they did from the last meeting, and then-- like, every time, like, what we did and then if there were problems and what we plan to-- like, exactly what was in the email, but we said it out loud so everybody could hear. And I think everybody-- it was quite helpful, I think. When they had a solution to-- or everybody said, oh yeah, that happened to me. Or-- it was like bonding. So, it was nice, and everybody got to speak, and it was encouraged. And I don’t think anybody was really quiet about it. So, I think everybody enjoyed sharing. Yeah, and it was nice to speak up and see if other people felt the same way sometimes.

(Lonely, mid-intervention)

I liked the meeting. I liked the fact that other people shared their difficulties. It makes me feel not so alone.

(Not lonely, post-intervention)

There are so many people out there in our age group that would benefit from this if they knew about it. It’s-- so many of us people in, you know, in their 60s feel uncomfortable going to a gym because it’s, you know, full of 20 year olds and you feel like you don’t belong. And this-- with our instructor it just made us feel like we were part of a group like everybody else. It was a good feeling.

Sharing/learning opportunities within Motivational Group Meetings were considered a more fruitful way to promote social connectedness/reduce loneliness than were ‘lecture’ style sessions.

(Not lonely, post-intervention)

But it might be nice to have actually had a-- even if it was just a get together with the group, just to see how everybody else did. I know that one lady was wanting-- she had joined the group with the purpose of, you know, finding someone else to exercise with. Which is a good thing too. But there wasn’t a lot of social opportunity, I think, because we got information. We were given-- there was a video and there was talks and exercises and discussions about things that you did individually. But we really didn’t have a lot of opportunities to sort of talk to one another. And that’s [inaudible] I think everybody’s fairly shy. But it may be something that they could throw in, maybe halfway and again at the end. A little social time, a tea or something. And just everybody could sort of talk about how they’re doing things. ‘Cause we learn from what some of the other people were doing too. So that was a good thing

*Engage with others who share similar/familiar experiences* promotes emotional and informational support to participants and offers space to share common characteristics or life experiences-- this fosters a sense of belonging and companionship.

(Lonely, baseline)

There were other people in the class that, when we all introduced ourselves, were having the same kind of struggles I had, the same kind of goals and were people that I thought, hmm, okay. There’s somebody I could probably call; see how they’re doing because they’re like me.

(Lonely, mid-intervention)

Well, companionship or-- communication and companionship with the other people who are attending. Yeah, these weren’t people that I knew prior. It just-- I think it’s just more supportive when there are other people that you’re hearing are dealing with issues too that are similar, um-hum.

*Increased opportunity for meaningful interaction*. Participants enjoyed the more frequent interactions during the active phase (first 3 months) of Choose to Move and felt the decline in motivational group meetings over the later months (taper phase) negatively influenced their sense of social connectedness.

(Lonely, mid-intervention)

Well, I think *name* (activity coach) did say they were continuing for three more months for checking in on us, right. And I’d assume it’s through email, right, that it wasn’t over. But it would have been nice, I think, to do it one last time, to end the program, just for a goodbye. I guess it’s because I like the group maybe too, yeah.

(Lonely, post-intervention)

It was nicer when we met more frequently, I think. I think that was-- yeah, ‘cause we met-- the first while we were meeting once a week, then once every two weeks and then it got to the month. I think the interaction for some people is a good thing. Through the winter that was really nice to have that group to go back to every few weeks, that other group, yeah. I think maybe a little more interaction would be good.

#### Check-ins

Participants described specific *Activity Coach characteristics/personality traits and approaches* that promoted a sense of social connectedness/ reduced loneliness during the Check-Ins.

*Activity coach characteristics/personality traits and approaches* that promoted social connectedness during check-ins included: being personable (easy to talk to), positive, someone who offers encouragement, accommodates, is accepting, motivates, provides accountability, approachable, high energy, makes sure to be available, takes time, listens, and is thoughtful.

(Lonely, mid-intervention)

Well, I think if they were serious about trying to get more physical in their activity and-- slowly and realistically and with support from the class and the instructor. And also, like, personal checks, either how would they prefer, email, face-to-face or phone call. So, I think it’s a really good follow-up, because a lot of times you get lost in the programs or it doesn’t seem like anybody cares, so you don’t care.

(Not lonely, mid-intervention)

Especially the encouragement. I mean, that’s the main thing anyway for me. ‘Cause I live alone and it’s easy to not do anything. So, it’s very nice when someone phones you up and says, how are you doing and, you know, can I help you in any way, get some ideas together and stuff like that. So that helps a great deal

(Not lonely, mid-intervention)

The phone calls are very encouraging. So that helps a lot. Yeah, she’s [activity Coach] fabulous. Really is a dear friend already so-- wonderful lady, and a very good encourager

*Accountability* promoted social connectedness by providing participants a sense of responsibility to the Activity Coach and to themselves and the other older adults in their group. The pre-planned check-in offered a consistent point of contact for participants that many looked forward to. Participants were accountable to the Activity Coach, which motivated and encouraged participants to engage in activity.

(Lonely, mid-intervention)

I mean, she’s right on top of it because she’ll make-- actually make an appointment for you. So that’s a good thing too, right. Because like I said, because I’m so busy doing stuff, that way I already-- it’s sort of pre-planned. I know she’s going to be phoning on that day, approximate time and all that. So, it’s not like I-- you know, so I already know that’s going to take place, and that’s great, yeah

(Lonely, post-intervention)

Well, I thought it was good. And she was excellent, and she made sure before she hung up that we had a date set and I had it written in my calendar. A date and time that she would call her next call. So, all the time when you’re-- if you weren’t doing something, in your head you know oh, you-- I’ve got to tell [name of Activity Coach] that I haven’t been doing anything. So, it’s just that little guilt trip there too, I guess

## Discussion

CTM influenced PA and loneliness differently among older adults who identified as lonely versus older adults who identified as not lonely. It is not clear within an array of intervention mechanisms which ones directly or indirectly influence intervention effectiveness [57]. Thus, we describe factors within components of a health promoting PA intervention (CTM), that were most likely to promote social connectedness/reduce loneliness. In doing so we heed the call to assess mechanisms that ‘move beyond the current focus on the objective social network as a way to promote social connectedness for older adults’ (pg.1) [12]. We also identify key indicators of social connectedness within these mechanisms that likely moderate this influence—a novel contribution to the literature. Our findings support the benefits of choice-based, group-focused interventions delivered to older adults in community settings.

It was telling that more than half (58%) of older adults in our study identified as lonely at baseline. In a recent systematic review [58], people described as more lonely were less physically active. We attribute the decline in PA in the lonely group during the last 3 months of the program to the reduced number of contacts with the activity coach during this ‘taper’ period. Lonely participants valued and expressed a desire for more motivational group meetings [59], as they fostered social connections (e.g., feeling cared for, belonging, meaningful relationships). At baseline more women (61%) than men (48%) identified as lonely. PA levels of women were more likely than men to be influenced by loneliness [24]. This speaks to gender sensitive implementation approaches; group, as compared with individual-based interventions may more effectively influence social connectedness in lonely women.

It is perhaps not surprising that lonely, as compared to not lonely participants, valued different parts of CTM program delivery. Activity coach characteristics/personality traits were the nexus of CTM program effectiveness—and especially valued by lonely participants. Activity coaches promoted social connectedness across all three CTM components. They were considered key to older adult participation [38] and CTM’s (phases 1 and 2) effect on mobility, social connectedness, loneliness and PA [37]. Specific characteristics/traits that influenced feeling socially connected were: being personable (easy to talk to), positive, accommodating, encouraging, accepting, observant, careful, motivating, and approachable. By identifying specific traits, we ‘drill down’ into meaningful aspects of how activity coaches respond to the needs and concerns of participants to generate feelings of social connectedness. For example, activity coaches called every participant by name so older adults got to know each other; they encouraged participants to share their experiences which cultivated a sense of bonding and belonging among the group. In traditional fitness classes the fitness leader role is more technical and prescriptive [60]. CTM activity coaches were less prescriptive serving more as a recreational ‘champion’ [61] by encouraging participants to do what they chose to do (and to stick with it). There is no one-size-fits all approach to addressing loneliness or physical inactivity in social connectedness and physical activity interventions; hence there is a key role for champions (like activity coaches) to tailor interventions to suit the needs of the individual participants [57]. CTM activity coach training included elements of social support and building a sense of community to enhance social connections--ideas that they embedded into their delivery approach. We were unable to find previous studies that described characteristics, skillsets and approaches of activity coaches that were likely to influence participant level outcomes---specifically, social connectedness [5].

In the motivational group meetings participants described the importance of sharing information and experiences, learning from others and engaging with others who shared similar/familiar experiences. Their perceptions distinguished between a group of older adults in a room receiving a ‘lecture’ about aspects of health versus embedding strategies that foster interaction and communication among the group. Oral or video presentations may reduce social isolation by increasing social contact, but may not generate feelings of being cared for, belonging or the development of meaningful relationships [12]. Social connectedness was facilitated when participants were partnered with others who shared similar experiences. In our study, shared experiences cultivated feelings of belonging (e.g., not alone, companionship) -- key indicators of social connectedness. Older adults may be more ‘comfortable’ and feel more ‘supported’ when exercising with others who are perceived to be similar to them [62].

Check-ins were instrumental to develop meaningful relationships between older adults and their activity coach. However, motivational group meetings were the core component that influenced social connectedness. Understanding the program components of CTM that drive effectiveness is essential to optimize interventions [63]. Optimization is defined as a “deliberate, iterative and data-driven process to improve a health intervention and/or its implementation to meet stakeholder-defined public health impacts within resource constraints” [63]. It may be prudent to adapt CTM to decrease the number of individual phone check ins (higher resource use) and increase the number of motivational group meetings (lower resource use). Future work (CTM phase 4; Fig. 1) will evaluate whether intervention effectiveness persists; if so, an optimized model would serve as one means to enhance social connectedness outcomes while also enhancing scalability and sustainability of CTM (through reduced cost).

To maintain benefits for individuals beyond the initial intervention, behaviour change must be maintained – a potentially challenging feat to achieve [64]. While evidence suggests that behaviour change is maintained beyond the end of an intervention for healthy inactive adults (≥18 yrs) [65], this does not appear to hold true for older adults in the absence of strategies designed to support maintenance [66]. Studies that formally evaluated effective strategies to maintain intervention-related benefits in older adults specifically are scarce [67]. Our findings demonstrate that lonely older adults value and desire increased and continued interaction with each other. A systematic review of adults ≥18 yrs. noted that maintenance strategies such as extended contact interventions and booster strategies to reinforce the initial intervention supported long-term effectiveness [68]. These took the form of a lower intensity intervention after a more intensive initial intervention [69], and booster sessions over the longer term that provide opportunities for groups to meet. Together these strategies may counter the known decline in lonely participants PA and social connectedness. We continue our efforts to optimize the costs, and sustain the benefits, of CTM during scale-up. We are currently evaluating the effectiveness of an optimized (reduced cost) ‘sustainability’ model on person-level outcomes.

### Limitations

We acknowledge volunteer and recruitment bias as the reach of partner organizations was primarily to a white middle-class (on average) constituency. Given our relatively homogeneous sample, results cannot be generalized to older adults who are marginalized by virtue of sex, gender, geography, socio-economic status, health status and/or ethnicity [37]. We randomly selected older adults to participate in interviews. However, as in our previous pre-post, hybrid effectiveness study, participants were not randomly assigned to group. In future, our findings should be replicated in a study purposely designed to evaluate the independent and combined effects of loneliness and social isolation on PA; future studies should include participants who identify as lonely but not isolated, isolated but not lonely, lonely and isolated, and neither lonely nor isolated. Conversely the direct or mediating effect of PA on loneliness and social isolation warrants further attention.

We classified participants as ‘lonely’ if they responded ‘some of the time’ or ‘often’ to any of the three questions on the questionnaire [70]. We acknowledge that this dichotomization may obscure variation within the ‘lonely’ category. However, a previous study noted similar findings when the ‘lonely’ category was further broken down into ‘moderately’ and ‘severely’ lonely groups [70].

Finally, we used a single item, self-report measure of PA as a means to reduce participant burden and to enhance feasibility of a province-wide evaluation. We acknowledge the potential for social desirability bias with self-report measures. In addition, although the single item PA questionnaire demonstrates acceptable reliability [44] and validity [45] the output (number of days per week over 30 min) does not capture all aspects of PA behaviours such as duration, intensity, type or domain [71]. Thus, we are unable to directly assess participants’ compliance to PA guidelines and may not capture all relevant changes in PA behaviours. Although there is a need for short, pragmatic tools for scale-up studies, there is a need for more nuanced PA questionnaires that ascertain the influence of loneliness on specific aspects of PA behaviours.

## Conclusions

First, given the ‘epidemic of loneliness’ that plagues many countries currently [5], PA and social connectedness interventions are timely and important. Although our study was not conducted in the COVID-19 environment, our findings have tremendous implications as COVID public health directives escalate social isolation and feelings of loneliness. Key factors of the CTM intervention that influenced social connectedness for older adults included interactions with the activity coaches, the opportunity to engage with other older adults and share information and experiences, and increased opportunity for meaningful interaction. CTM participants who were lonely reduced their PA during the last 3 months of the program (taper phase). We attribute this to fewer contacts between older adults and their activity coach. Second, health promoting interventions that were effective at small scale must be scaled up to promote physical, social and mental health at the population level. Strategies to scale-out CTM—'implement, test, improve, sustain and optimize an evidence-based intervention delivered to new populations and/or through new delivery systems that differ from those in effectiveness trials’ [71] —are in order. Third, to honor the central tenets of equity, diversity and inclusion, health promoting flexible programs like CTM should be adapted for older adults who are marginalized by virtue of their sex, gender, geography, socio-economic status, health status and/or ethnicity.

## Data Availability

The datasets used during the current study are not publicly available as stipulated in our participant consent forms but are available from the corresponding author on reasonable request.

## Abbreviations

BC: British Columbia
COVID-19: Coronavirus disease
CTM: Choose to Move
PA: Physical activity
